# Efficacy and safety of electric heating moxibustion for perennial allergic rhinitis: protocol for a randomized controlled trial

**DOI:** 10.1186/s13063-019-3550-x

**Published:** 2019-07-19

**Authors:** Chan-Yung Jung, Min-Jin Cho, Ha-Ra Kang, Seung-Ug Hong, Won-Suk Sung, Eun-Jung Kim

**Affiliations:** 1 Institute of Oriental Medicine, College of Korean Medicine, Dongguk University, Gyeongju, South Korea; 2 College of Korean Medicine, Dongguk University, Gyeonggi-do, South Korea; 3 Department of Acupuncture and Moxibustion, Dongguk University Ilsan Oriental Hospital, Gyeonggi-do, South Korea; 4 Department of Ophthalmology, Otolaryngology and Dermatology, Dongguk University Ilsan Oriental Hospital, Gyeonggi-do, South Korea; 5 Department of Acupuncture and Moxibustion, Dongguk University Bundang Oriental Hospital, Gyeonggi-do, South Korea

**Keywords:** Allergic rhinitis, Electric heating moxibustion, Moxibustion, Randomized controlled trial

## Abstract

**Background:**

Allergic rhinitis (AR) is an IgE-mediated disease that adversely affects quality of life. Many studies report that moxibustion is an effective treatment for perennial allergic rhinitis (PAR). However, it is difficult to perform moxibustion on the face because of possible burning of the skin and the noxious effects of smoke. Electric heating moxibustion does not have these limitations. The purpose of this clinical trial is to assess the possibility of treating PAR with electric heating moxibustion and to assess the feasibility of conducting a clinical test on a larger scale.

**Methods:**

This is a randomized, open-label, assessor-blind, parallel-design pilot clinical study. We will recruit 40 eligible participants and randomly allocate them into an electric heating moxibustion group or an acupuncture group at a 1:1 ratio. Patients in both groups will receive eight treatments over 4 weeks, and the final follow-up will be 4 weeks after the last treatment. Eleven acupuncture points will be used for patients in both groups (EX-HN3 and bilateral EX-HN-8, LI20, LI4, GB20, and ST36). The primary outcome measure is change in the Total Nasal Symptom Score, and the secondary outcome measures are changes in the Rhinoconjunctivitis Quality of Life Questionnaire, nasal endoscopy index for pattern identification, pattern identification questionnaire for AR, total IgE, eosinophil count, and adverse effects.

**Discussion:**

This clinical trial will examine the effect of electric heating moxibustion on PAR.

**Trial registration:**

ClinicalTrials.gov, NCT03342105. Registered on 14 November 2017.

**Electronic supplementary material:**

The online version of this article (10.1186/s13063-019-3550-x) contains supplementary material, which is available to authorized users.

## Background

Allergic rhinitis (AR) is a highly prevalent disease that causes physical, psychological, and social problems in daily life [1]. About 10% of the world’s population experience seasonal allergic rhinitis (SAR), and about 10–20% have perennial allergic rhinitis (PAR) [2]. Symptoms of PAR are present in 25% of pre-school children and 75% of school-age children in Korea [3]. The prevalence of PAR seems to have increased in recent years. The prevalence of symptoms related to AR is as high as 31.5% in the USA [4], and a survey of five countries in Europe reported the prevalence of AR was 22.7% [5]. These results thus indicate a high prevalence of AR worldwide.

AR is immunoglobulin E (IgE)-mediated inflammatory response, which can be subdivided into SAR and PAR. The former is caused by seasonal allergens (pollen from *Parietaria*, *Ambrosia*, *Artemisia*, and *Cupressus*) and the latter by perennial allergens (dust mites, animals, occupational factors) [1]. There is some evidence that acupuncture and moxibustion are effective treatments for PAR [6].

Conventional treatment of AR includes avoidance therapy, pharmacotherapy, immunotherapy, and surgery, although avoidance therapy is difficult in clinical practice [7]. Clinicians commonly treat patients with PAR using decongestants, antihistamines, steroids, and antileukotrienes [8]. However, there are limitations to these conventional treatments—no drug can cure PAR, and these treatments have reduced efficacy when taken for a long time and may also cause adverse effects [9]. Thus, there is a need for novel therapies that do not have these limitations [10].

Practitioners of traditional Korean medicine (TKM) have reported the efficacy of acupuncture [11], moxibustion [12], and Korean herbal medicines [13] on AR through various studies. Acupuncture is proven to exhibit anti-inflammatory actions [14] and regulate mediators such as interleukin (IL)-10 [15] and IgE [16]. Moreover, complementary DNA (cDNA) microarray analysis suggested acupuncture treatments as a modulator on the balance between pro- and anti-inflammatory cytokines [9].

Moxibustion is a TKM that aims to prevent and treat diseases by burning of herbal substances at acupuncture points, a process leading to thermal stimulation [17]. From the perspective of TKM, moxibustion treats diseases by encouraging Qi and blood circulation through thermal stimulation and thereby improves the function of internal organs. Various articles have suggested that moxibustion is effective for osteoarthritis-induced pain [18] and chronic fatigue [19] as well as AR [20].

However, conventional moxibustion can cause several adverse effects, especially when used to treat AR. First, facial burning is a potential danger because it is difficult to precisely regulate the temperature of the burning moxibustion [21]. Second, moxibustion releases particulate matter less than 10 μm in diameter (PM10), which is up to five times higher than the World Health Organization (WHO) standard [22], and this may actually exacerbate the AR [23]. Although acupuncture points on the head and face are often used to treat PAR, use of these points for moxibustion may trigger burns and respiratory problems. For these reasons, TKM and other alternative medicines have preferred acupuncture to moxibustion [24]. However, certain medical devices, such as electric heating moxibustion, allow the use of moxibustion without the adverse effects of conventional moxibustion.

This clinical trial will assess the possibility of treating PAR by electric heating moxibustion treatment and assess the feasibility of conducting a clinical test on a larger scale.

## Methods/design

### Objective

This study consists of a parallel clinical test on two groups of patients with PAR, treating one group with electric heating moxibustion and the other with acupuncture. As a pilot study, it seeks to assess the possibility of treating PAR with electric heating moxibustion and the feasibility of conducting a clinical test on a larger scale.

### Hypothesis

The null and alternative hypotheses are the following:

- H0: μ1 − μ2 = 0 (the null hypothesis, H0). There is no difference in the mean change of the Total Nasal Symptom Score (TNSS) before and after treatment between the moxibustion group and the acupuncture group. This will be tested versus the alternative hypothesis.
- H1: μ1 − μ2 ≠ 0 (the alternative hypothesis, H1). There is a difference in the mean change of the TNSS before and after treatment between the moxibustion group and the acupuncture group.

The objective is to test whether there is a difference between the mean change of the moxibustion group and the acupuncture group.

### Design and setting

This is a randomized, open-label, assessor-blind, parallel pilot clinical study. This trial will be performed at Dongguk University Ilsan Oriental Hospital in Korea. A total of 40 participants who meet the inclusion and exclusion criteria will be divided into two groups with a 1:1 allocation ratio. The control group will receive acupuncture, and the experimental group will receive electric heating moxibustion. The 8-week study period consists of a 4 week treatment phase and a 4 week follow-up phase.

**Fig. 1:**
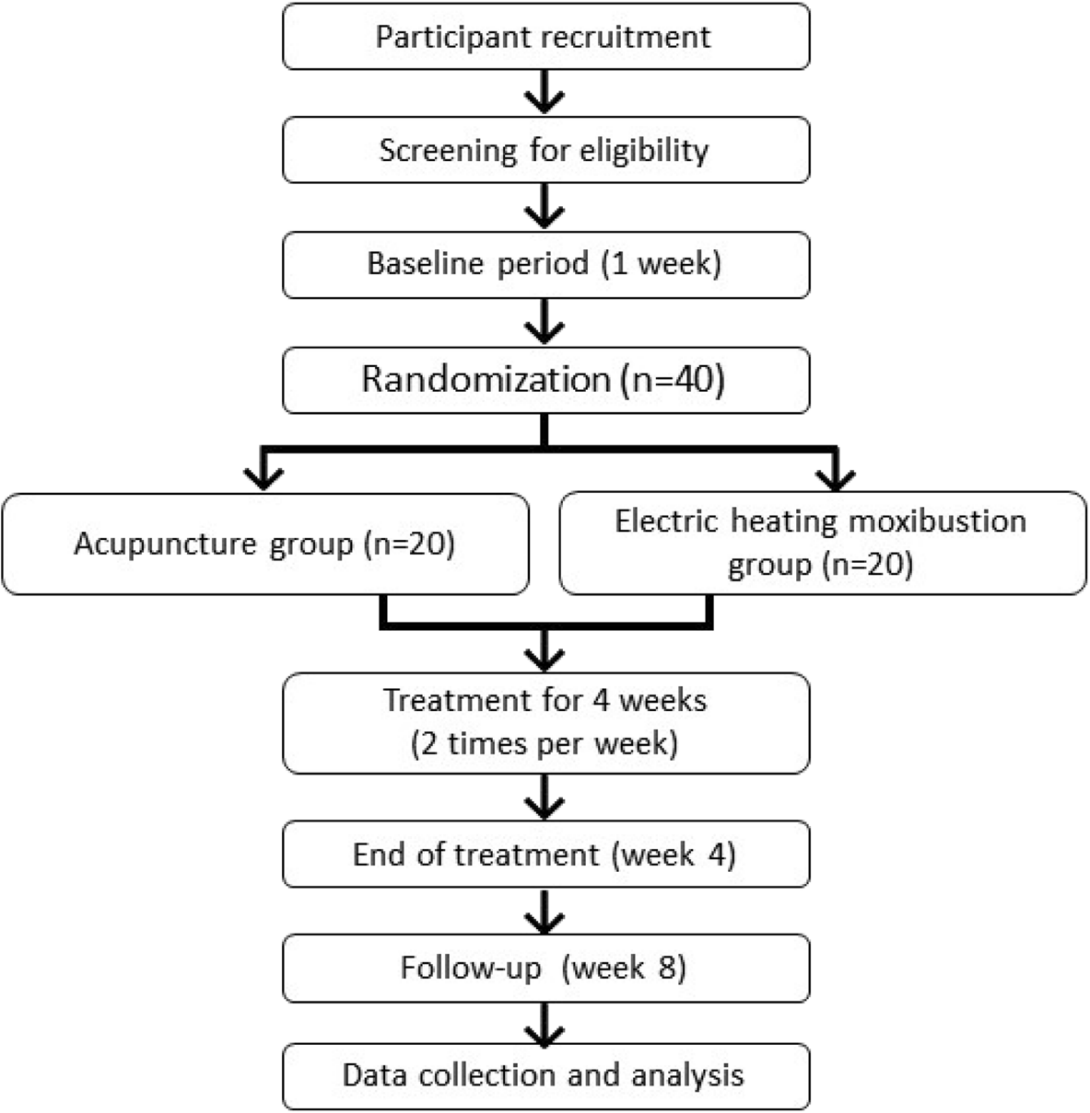
Flowchart of this study

**Fig. 2:**
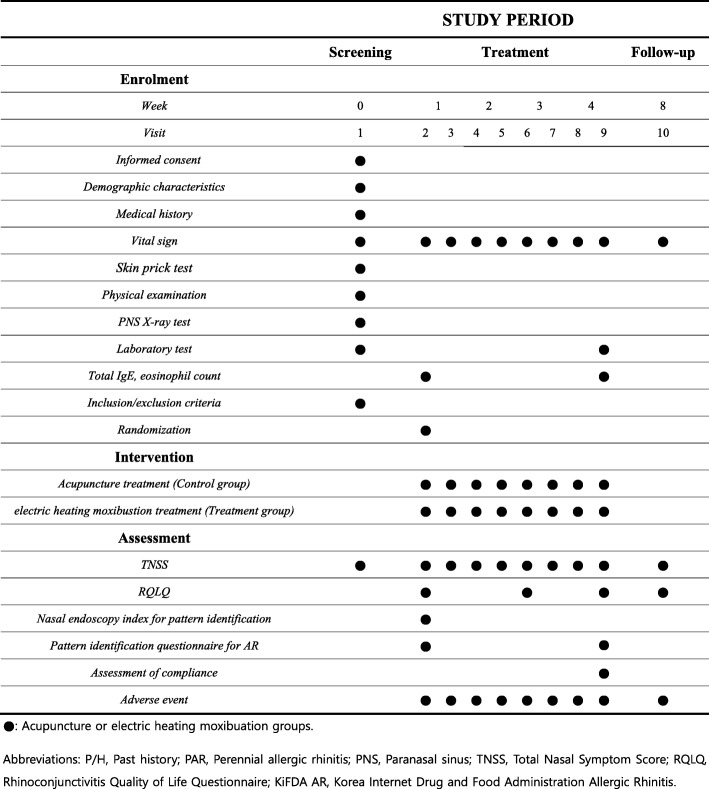
Schedule of enrollment, intervention, and assessments

Figure 1 outlines the trial procedures, and Fig. 2 shows the schedule for enrollment, intervention, and assessments. The Standard Protocol Items: Recommendations for Interventional Trials (SPIRIT) checklist is provided in Additional file 1.

### Study subjects

#### Inclusion criteria

The trial inclusion criteria are:

1. Presence of nasal symptoms for 2 successive years
2. Presence of at least two nasal symptoms (rhinorrhea, nasal obstruction, nasal itching and sneezing) with severity score of 2 or more (0: no symptoms, 1: mild symptoms, 2: moderate symptoms, and 3: severe symptoms)
3. Testing positive for at least one perennial allergen in a skin prick test (*Dermatophagoides farinae*, *Dermatophagoides pteronyssinus*, dog fur, cat fur, *Alternaria tenuis*, *Aspergillus fumigatus*, or cockroach) [25]
4. Age of 19 to 60 years old
5. Able to voluntarily agree to participate and to sign informed consent
6. Willingness to participate and sign the informed consent agreement.

#### Exclusion criteria

The exclusion criteria are as follows:

1. Use of drugs that may directly affect AR or skin prick test results; use of nasal/oral corticosteroids within the past month; use of an herbal medication for rhinitis within the past month; use of nasal cromolyn or a tricyclic antidepressant within the past 2 weeks; use of nasal/oral decongestants, nasal/oral antihistamines, or antileukotrienes within the past week; use of drugs the researchers believe are inappropriate
2. Presence of rhinosinusitis, indicated by mucosal thickening or opacification of the paranasal sinuses, as indicated by a paranasal sinus X-ray
3. Presence of a malignant cancer, severe systemic inflammation, or other systemic disease that may affect rhinitis
4. History of anaphylaxis in response to allergy tests
5. Females who are pregnant or lactating (positive reaction to the human chorionic gonadotropin [hCG] test)
6. Having difficulty in maintaining treatment due to paralysis, a severe physical or psychiatric disorder, dementia, drug addiction, or severe visual or audiogenic impairment
7. Being afraid of electric heating moxibustion or of the expected adverse effects
8. Conditions which make research participation logistically impossible, such as lack of ability or willingness to make time to participate, to make regular visits to the hospital, or to engage in oral and written communication in Korean.

### Sample size

In the recent systematic reviews on rhinitis with acupuncture and moxibustion, there was no study that used moxibustion alone on PAR [26]. Furthermore, due to the special method of using electric moxibustion, we could not utilize the existing research, and it was necessary to have a flexible approach to statistical design with an exploratory study.

According to the suggestion of Julious [27], the appropriate sample size for a two-arm pilot study should be more than 12. Thus, considering the possibility of dropouts and the other characteristics of this pilot study, we will initially recruit 20 individuals per group.

### Recruitment

Participants will be recruited via outpatient and inpatient clinical recruitment posters. Printed recruitment posters will be distributed in hospital and placed on bulletin boards and websites. The posters will contain a brief introduction to the trial, the details of treatments, and contact information. Participants who wish to participate can directly contact the researcher.

Potential participants will be screened, receive explanations of the trial, and then sign informed consent agreements. Those who meet the selection criteria will receive baseline assessments, and demographic and general medical data will be collected, including medical history, vital signs, skin prick test, physical examination data, radiographic data, and laboratory data to determine eligibility.

### Randomization and blinding

Randomization will be achieved with a computerized random number generator using the stratified block randomization method of SAS (SAS Institute, Inc., Cary, NC, USA). This will be performed by an independent statistician who is not involved in the clinical trial.

The random numbers will be concealed using sequentially numbered, opaque, sealed envelopes. The participant’s envelope is delivered to the site’s code manager. If a participant is enrolled and needs randomization, the envelope will be sent to the TKM doctor who performs the treatment. The doctor will open the envelope in front of the participant. A list of generated codes and SAS programs is kept by statisticians in case of loss.

### Interventions

**Fig. 3:**
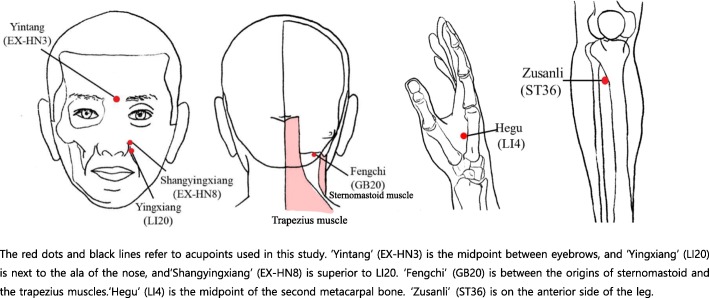
Acupoints used in this study

The treatment will be performed in eight sessions, two times per week for 4 weeks. Follow-up assessment will be conducted at 4 weeks after the end of treatment. Each treatment session will be 15 min in duration. The patients in each group will receive treatment at 11 standard acupuncture points: Yintang (EX-HN3), and bilateral Shangyingxiang (EX-HN-8), Yingxiang (LI20), Hegu (LI4), Fengchi (GB20), and Zusanli (ST36) (Fig. 3). The treatment acupuncture points were selected based on a consensus of TKM doctors, a textbook, literature reviews, and additional studies [28, 29].

#### Electric heating moxibustion

**Fig. 4:**
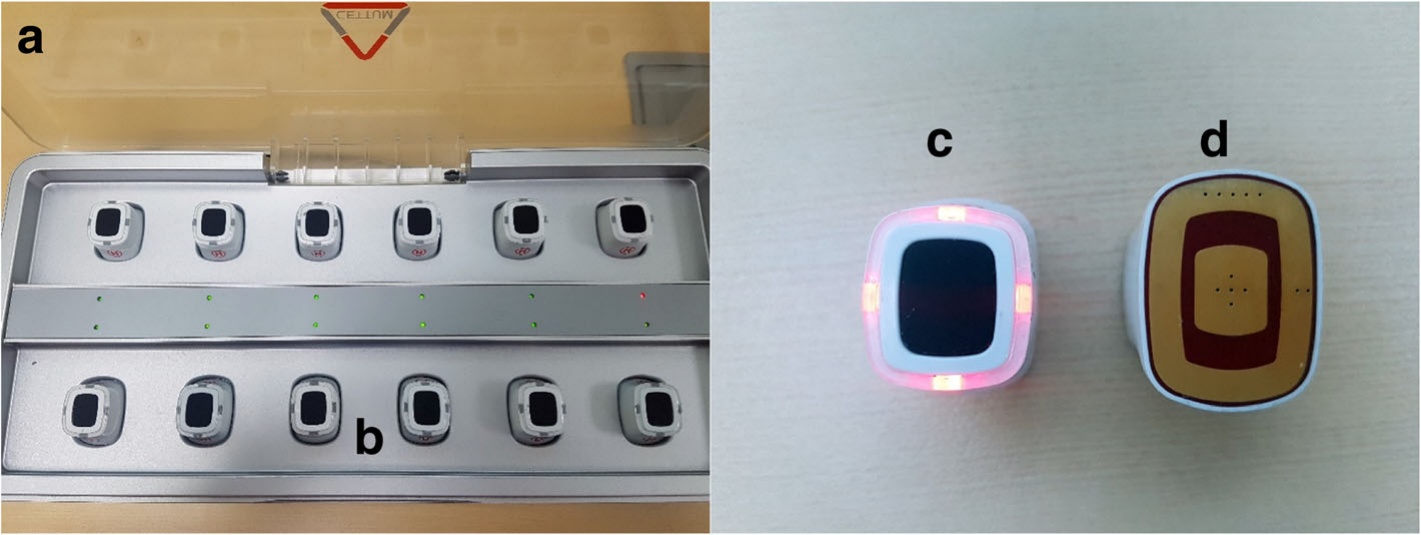
**a** Electrical heating moxibustion device, **b** the charging equipment, **c** the power unit, **d** the bottom of the unit

The electric heating moxibustion will be performed at 11 acupuncture points twice per week for 4 weeks using a specially designed device (Cettum, K-Medical Co., Korea) (Fig. 4).

The electric heating moxibustion device has two parts: the heating units and the charging equipment. The practitioner will attach the heating units to the patient’s skin, and the temperature will be set automatically at 10 s after pressing the button on top of the heating unit, as indicated by a change in color of the light-emitting diode (LED) on the button. The temperature will increase to 45 ± 1 °C, remain stable, and then gradually decrease. If a participant complains of unbearable pain or hotness, the attached point will be moved 1 cm or less, based on the judgment of the practitioner.

#### Acupuncture treatment

Acupuncture will be performed at 11 acupuncture points twice per week for 4 weeks using disposable and sterile acupuncture needles (0.25 × 30 mm) (Dongbang Co., Korea).

The guidelines for acupuncture treatment are as follows: the needles will be inserted to a depth of 2–3 mm, perpendicular to the skin surface; there will be no De Qi or reinforcing-reducing manipulation for acupuncture points on the face; and reinforcing-reducing manipulation will be performed by twirling the needle for Hegu (LI4) and Zusanli (ST36).

#### Combined use of medications

Surgical interventions, drugs that may directly affect AR or skin prick test results (nasal/oral corticosteroids, nasal cromolyn, tricyclic antidepressants, nasal/oral decongestants, nasal/oral antihistamines and antileukotrienes), traditional Korean medical treatments (such as acupuncture and herbal medicine to relieve symptoms of AR), newly administered psychotropic drugs, or narcotic analgesics will not be allowed.

Medications that were taken before trial participation will be allowed if the investigators believe they will not affect the interpretation of the outcome. Medications used temporarily for treating new diseases or adverse effects will be allowed pending consultation between the researchers. Detailed information on medications will be recorded in the case report form (CRF).

At the discretion of the physician responsible for the treatment, and based on the medical condition of the subject, if a subject uses a non-allowed medication for treatment during the trial, the subject will be excluded, and this will be recorded in detail on the last page of the CRF.

### Quality assurance

Participants will receive the acupuncture and moxibustion treatment by TKM doctors who satisfy the following requirements:

1. Graduation from a 6-year, full-time course in TKM, taught as a college program
2. Certification by the Korean Ministry of Health and Welfare as a TKM doctor
3. More than 1 year of postgraduate clinical training in a Korean medicine hospital
4. Completion of the first-year residency program in the Department of Acupuncture and Moxibustion at Dongguk University.

Nasal endoscopy and pattern identification will be assessed by TKM doctors who specialize in ophthalmology and otolaryngology.

To reduce bias, the assessor not involved in the randomization and treatment will perform outcome assessment in a separate room from the treatment room.

The practitioners of acupuncture and electric heating moxibustion will receive training on the diagnosis of PAR, the inclusion and exclusion criteria, standard operation procedures, location of the acupuncture points, manipulation techniques of the devices used for electric heating moxibustion and acupuncture, and outcome measures.

### Outcome assessments

#### Primary outcome

The primary outcome measure will be the change of the Total Nasal Symptom Score (TNSS) from baseline (visit 2) to the end of treatment (visit 9). The TNSS evaluates each of four symptoms (rhinorrhea, nasal obstruction, and nasal itching and sneezing) on a 4-point scale (0: no symptoms, 1: mild symptoms, 2: moderate symptoms, 3: severe symptoms], so the total score ranges from 0 to 12 [30].

#### Secondary outcomes

The secondary outcomes are changes in the Rhinoconjunctivitis Quality of Life Questionnaire (RQLQ) score, nasal endoscopy index for pattern identification, pattern identification questionnaire for AR, total IgE, eosinophil count, and the presence of adverse effects. These measures were selected to compare the efficacy, adverse events, and safety of the two treatments, according to Korean Medicine pattern identification.

##### Rhinoconjunctivitis Quality of Life Questionnaire (RQLQ)

The RQLQ is a self-reported questionnaire used to assess the quality of life in patients with AR. The questionnaire has seven domains: activities, sleep, practical problems, nasal symptoms, eye symptoms, emotions, and non-hay fever symptoms [31]. Patients will be asked to recall their experiences during the previous week and to rate each answer using a 7-point scale (0: no impairment, 6: severe impairment). The RQLQ will be administered during visits 2, 6, 9, and 10.

##### Nasal endoscopy and pattern identification

These measures consist of an index and a questionnaire:

1. *Nasal endoscopy index for pattern identification*. The investigator (a TKM doctor) will evaluate the nasal endoscopy index during visits 2 and 9. The investigator will determine the score based on observations of the nasal membrane color, the presence of rhinorrhea, and the presence of inferior turbinate swelling (using nasal endoscopy) [32].
2. *Pattern identification questionnaire for AR*. The investigator (a TKM doctor) will select a pattern for each patient (Lung Heat, Lung Cold, and Spleen Qi Deficiency) by face-to-face diagnosis and based on body and nasal conditions [33].

##### Total IgE, eosinophil count

Total serum IgE and eosinophil count levels will be measured during visits 2 and 9.

##### Adverse effects

Any unexpected symptoms will be checked at every visit, and their time of occurrence and duration will be recorded.

### Adverse events

Previous studies reported that common adverse events caused by moxibustion were blister, redness, itching, burns, keloids, discoloration, and allergic reaction to the tape [34]. Common adverse events caused by acupuncture were bruising, peripheral neuritis, cellulitis, allergic reaction, pain lasting more than 2 weeks, and dermal hypersensitivity [34].

At every visit, the participants will report adverse events, and the investigator will record the details, including the specific symptom, onset, duration, severity, time of resolution, and possible association with treatment. The practitioner will decide whether to pause treatment, depending on the severity, to prevent exacerbation. The practitioner will also measure vital signs at every visit.

### Dropout

Participants who meet any of the following criteria will be excluded from the trial, and the specific reasons will be fully recorded: withdrawal of consent; new surgical intervention, injection, or oral drug for treatment of rhinitis during clinical trial period; receiving fewer than six treatment sessions or not participating in the follow-up; experiencing severe adverse events making further inclusion in the trial unsustainable; and if the researcher determines that further participation is inappropriate.

### Withdrawal and discontinuation

Participants can withdraw voluntarily at any time during the trial. Participants who are unable to complete the study, regardless of time or reason, are considered dropouts. The last recorded data for these participants will be included in the data analysis.

Withdrawal due to adverse events is distinguished from withdrawal due to inadequate response. All adverse effects are analyzed at the study endpoint regardless of whether they are considered relevant to the study treatment or not. If a participant withdraws due to a serious adverse event, it will be reported according to the reporting requirements. Where appropriate, data collected by these participants will be used in intention-to-treat analyses.

### Data management and monitoring

Study data will be collected in the CRF by clinical research coordinator (CRC) who carried out a Good Clinical Practice training course. No record will be missed or omitted, and the primary input of the data will not be permitted to be changed. Any corrections should be explained in the appended notes signed and dated by the researcher.

For data confidence, written data will be stored in a locked space at the study sites with access limited to the researchers. Electronic data will be stored in a password-protected computer.

Data will be double-entered. Double data entry of CRFs will be conducted by two experienced independent data entry clerks within 2 weeks of data collection. The data stored in a finalized clinical trials database will be ensured to be an accurate reflection of its source and will conform to specific standards of quality.

The study monitoring will follow Good Clinical Practice principles and will be processed by the Korean Medicine Clinical Trial Center of Kyung-Hee University. No formal data monitoring committee will be convened for this study. However, a clinical research associate (CRA) will be in attendance every 4 weeks to monitor and ensure the quality of the recorded data. The CRA will check the medical records, informed consent forms, source documents, and the CRFs.

### Statistical analysis

An independent statistician blinded to group allocation will perform statistical analysis on the full analysis set (FAS) and will also perform a per-protocol (PP) analysis. Missing data imputation (last observation carried forward [LOCF]) will be used to evaluate the robustness of the primary endpoint. The null hypothesis is that the two groups have no changes in any of the outcome variables.

The independent-samples *t* test or the Mann-Whitney test will be used to compare the primary outcomes between the two groups. For the primary outcome measure, a repeated measures analysis of variance (ANOVA) will also be used to test the effect of time and the treatment cross-effect.

For secondary outcome measures, the independent-samples *t* test or the Mann-Whitney test will be used to analyze continuous variables, and the chi-squared test to analyze categorical variables. For safety outcomes, the incidence of adverse events will be determined, and the two groups will be compared. A *P* value below 0.05 will be considered statistically significant.

### Ethics

The protocol complies with general ethical guidelines (the Declaration of Helsinki and Korean Good Clinical Practice), was approved by the institutional review board of Dongguk University Ilsan Oriental Hospital, and was registered at ClinicalTrials.gov (NCT03342105). Prior to onset, all participants will be informed of the methods of the trial, including possible benefits and adverse events, and their responsibilities. All enrolled subjects will voluntarily enter the study and will provide written informed consent prior to participation.

## Discussion

AR is a common disorder that interferes with the daily activities and reduces the quality of sleep of an affected individual, and it is also a significant societal burden [5]. We chose to study individuals with PAR because generally symptoms of PAR are more severe than those of SAR [35] and because SAR can evolve into PAR [7], as symptoms worsen following exposure to seasonal and perennial allergens.

Nasal itch, sneezing, rhinorrhea, and nasal obstruction, which are mediated by an elevation of IgE and mast cells, are the most common symptoms of AR. Thus, when a patient is exposed to an allergen, such as house dust mites, cockroaches, mold, or animal dander, IgE-sensitive mast cells degranulate and upregulate leukotrienes, cytokines, histamine, and prostaglandins, leading to the symptoms of AR [36].

Previous research reported that acupuncture and moxibustion are often effective treatments for AR [20, 37]. In fact, both treatments had been used thousands years ago, and WHO also listed 43 acupuncture and moxibustion-treated diseases, which included those of the respiratory system [38]. Moxibustion is believed to work by providing heat and chemical stimulation [39]. In TKM, AR is usually attributed to a deficiency of Wei Qi and wind and cold [40]. In agreement, modern medicine indicates that AR can be caused or aggravated by the cold air that occurs during winter and in the early morning [41]. The heat stimulation provided by moxibustion may thus account for its efficacy in treating AR [40]. Moxibustion also functions in immunomodulation through immunoglobulin, cytokines, and immune organs like the thoracic duct and spleen [42]. In particular, moxibustion can reduce the levels of interleukin (IL)-4, increase the interferon (IFN)-γ/IL-4 ratio, and reduce IgE-mediated inflammatory reactions [43].

Although moxibustion can provide clinical benefit to patients with AR, it is difficult to perform on the face due to the risk of burns and smoke inhalation. A recent study examined 814 patients who received TKM for AR and found that 72% received acupuncture but only 0.2% received moxibustion [24].

Electric heating moxibustion was recently developed to avoid the disadvantages of conventional moxibustion while maintaining its clinical benefits. The electric heating moxibustion device can automatically stop increasing the temperature when it reaches a critical level, thereby preventing burns and fire. In addition, electric heating moxibustion does not generate smoke or fine dust and thus does not cause respiratory discomfort. Electric heating moxibustion is also easier to implement and does not require an ignition system or an exhaust system. Most importantly, electric heating moxibustion can maintain an appropriate temperature and produce the same thermal stimulus as traditional moxibustion [44]. These many advantages are the reasons we assess the possibility of treating PAR with electric heating moxibustion.

There are several limitations of this trial. First, the number of participants (total 40) is insufficient for a clinical trial. However, our purpose at this time is to check the feasibility of using electric heating moxibustion for treating PAR. Second, it is difficult to blind both the practitioner and the participants, because the instruments used for acupuncture and electric heating moxibustion are very different. Therefore, we will attempt to reduce the bias by blinding an assessor who does not participate in the patient allocation assignment or the administration and practice of the treatment. Despite these limitations, this will be the first clinical study to examine the use of electric heating moxibustion for the treatment of AR.

### Trial status

The final protocol version is 1.3, dated 17 May 2018. The recruitment began on 23 May 2018 and is ongoing.

## Additional file

Additional file 1: SPIRIT 2013 checklist. (DOCX 52 kb)

## Data Availability

Data and material from this trial are available upon reasonable request and approval by the corresponding author.

## Abbreviations

ANOVA: Analysis of variance
AR: Allergic rhinitis
CRA: Clinical research associate
CRF: Case report form
FAS: Full analysis set
ITT: Intention-to-treat
LOCF: Last observation carried forward
PAR: Perennial allergic rhinitis
PP: per-protocol
RQLQ: Rhinoconjunctivitis Quality of Life Questionnaire
SAR: Seasonal allergic rhinitis
TKM: Traditional Korean medicine
TNSS: Total Nasal Symptom Score
